# Preventive efficacy of a topical combination of fipronil – (S)-methoprene – eprinomectin – praziquantel against ear mite (*Otodectes cynotis*) infestation of cats through a natural infestation model

**DOI:** 10.1051/parasite/2014042

**Published:** 2014-08-25

**Authors:** Frédéric Beugnet, Émilie Bouhsira, Lénaïg Halos, Michel Franc

**Affiliations:** 1 Merial 29 avenue Tony Garnier 69007 Lyon France; 2 Parasitologie, École Nationale Vétérinaire de Toulouse 23 Chemin des Capelles 31076 Toulouse France

**Keywords:** Cats, *Otodectes cynotis*, ear mite, prevention, Broadline^®^

## Abstract

A study based on naturally infested cats was designed to evaluate the effectiveness of a single treatment with a topical formulation containing fipronil, (S)-methoprene, eprinomectin and praziquantel, for the prevention of *Otodectes cynotis* infestation in cats. Six treated cats and six untreated cats were housed with three chronically *Otodectes cynotis*-infested cats, respectively. The cats of each group were kept together in a 20-m^2^ room for 1 month. Both clinical examination and ear mite counts were conducted on Day 28. All donor cats were confirmed to be chronically infested with *Otodectes cynotis* on Day −1 and Day 28. From untreated control cats, 129 live mites were recovered on Day 28 and all cats were found to be infested. In the treated group, three cats were found to be infested, with a total of five live mites recovered, the difference between the two groups being significant (*p* = 0.003). One treatment corresponded to 96% preventive efficacy at Day 28 based on ear mite counts. With regard to cerumen, the clinical score increased significantly for untreated cats between Day −1 and Day 28 (*p* = 0.00026) and not for treated cats (*p* = 0.30). The difference in cerumen abundance was significant between untreated and treated cats on Day 28 (*p* = 0.0035). Concerning the pruritic reflex in at least one ear, all cats were negative at inclusion. All six untreated cats became positive and showed a reflex on Day 28, whereas no treated cat showed ear pruritus (*p* = 0.00026).

## Abstract (fr)

Une étude basée sur des chats naturellement infestés a été conçue pour évaluer l’efficacité d’un traitement unique par une formulation topique contenant fipronil, (S)-méthoprène, éprinomectine et praziquantel pour la prévention de l’infestation par *Otodectes cynotis* chez les chats. Six chats traités et six chats non traités ont été mis en contact, respectivement, avec 3 chats chroniquement infestés par *Otodectes cynotis*. Les chats de chaque groupe étaient maintenus ensembles dans une pièce de 20 m^2^ durant un mois. Une évaluation clinique ainsi que la collecte des acariens ont été faites à J28. Tous les chats donneurs ont été confirmés comme porteurs d’*Otodectes cynotis* aux jours J-1 et à J28. Dans le groupe contrôle, 129 acariens vivants ont été récupérés et tous les chats étaient infestés à J28. Dans le groupe traité, 3 chats ont été trouvés infestés et un total de 5 acariens a été retrouvé. La différence entre les groupes étaient significative (*p* = 0.003). Le traitement a permis d’obtenir une efficacité préventive de 96 % à J28 sur la base des comptages parasitaires. En ce qui concerne le cérumen, le score clinique entre J-1 et J28 était significatif pour le groupe contrôle (*p* = 0.00026) et pas pour le groupe traité (*p* = 0.30). La différence de l’abondance de cérumen était significative entre les deux groupes à J28 (*p* = 0.0035). Concernant le réflexe de prurit dans au moins une oreille, tous les chats étaient négatifs à l’inclusion. Les 6 chats du groupe contrôle sont devenus positifs et montrait un réflexe à J28 alors qu’aucun chat du groupe traité n’avait de prurit (*p* = 0.00026).

## Introduction

The ear mite, *Otodectes cynotis* (Hering, 1838), is one of the most common ectoparasites of cats [3, 6]. It is pathogenic in young cats and dogs, and directly transmitted from one animal to another [9, 10]. Several curative treatments are available, mainly including the use of a topical endectocide based on moxidectin or selamectin [1]. What is unknown is the preventive effect that could be obtained regarding direct transmission by infested cats. An experimental model based on naturally infested cats was designed to evaluate the effectiveness of a single treatment with a new topical formulation containing fipronil, (S)-methoprene, eprinomectin and praziquantel (Broadline^®^ spot-on, Merial) for the prevention of *Otodectes cynotis* infestation in cats [12].

## Materials and methods

This study was a parallel group design, randomized, controlled efficacy study (Table 1). It included two groups, each of six cats, plus three chronically *Otodectes cynotis*-infested cats used as donors. The infestation of donors was checked by otoscopic examination but no mites were removed, 2 weeks before the start of the study. No cat was treated with antiparasitic drugs in the 2 months before the start. At Day −1, the infestation status of all cats was checked by visual otoscopic examination (Tables 2 and 3).

**Table 1.** Design of the study.

Study day or range	Event
Day −7 to Day 0	Randomly allocate the 12 naive cats to group 1 or 2 Put each group into a specific large room
Day −1	Control the infested status of 6 ear mite-infested “donor cats”
Day 0	Treat group 2 cats
Days 7, 14, 21	Clinical examination of the cats to be sure of their healthy status and that they do not need treatment.
Day 28	All cats Clinical scoring* Complete ear flush for all cats followed by ear mite counts

* Clinical scoring system. Ear pruritus: 0 = absent; 1 = present and very low; 2 = mild; 3 = pronounced. Cerumen: 0 = absent; 1 = present but clear and with a low volume; 2 = moderate volume and brown color; 3 = abundant dark cerumen.

**Table 2.** Ear mite infestation and clinical status in donor cats.

Day −1	Sex	Right ear				Left ear			
Donors		Cerumen	Ear pruritus	Parasites		Cerumen	Ear pruritus	Parasites	
In group 1				Macroscopic*				Macroscopic	
3812	M	3	1	1		3	1	1	
6529	M	3	0	1		3	0	1	
4644	F	2	2	0		1	3	1	
In group 2	Average	3	1	3 cats		3	1.33	3 cats	
3996	F	3	3	1		3	1	1	
6573	M	3	1	1		3	1	1	
6502	F	3	1	1		3	1	1	
	Average	2.67	1.67	2 cats		2.33	1	3 cats	
Day 28	Sex	Right ear				Left ear			
Donors		Cerumen	Ear pruritus	Parasites		Cerumen	Ear pruritus	Parasites	
In group 1				Macroscopic*	Microscopic°°			Macroscopic	Microscopic°°
3812	M	3	3	1	10	3	2	1	9
6529	M	3	3	1	30	3	3	1	16
4644	F	3	3	1	31	3	2	1	928
In Group 2	Average	3	3	3 cats	**71**	3	2.67	3 cats	**953**
3996	F	3	3	1	26	3	2	1	102
6502	F	3	2	1	10	3	0	1	5
6573	M	3	2	1	373	3	2	1	899
	Average	3	2.33	3 cats	**409**	3	2.67	3 cats	**1006**

Cerumen: 0 = absent; 1 = present but clear and low volume; 2 = moderate volume and brown; 3= abundant dark.

Ear pruritus reflex: 0 = absent, 1 = present and very low, 2 = mild, 3 = pronounced.

* Parasites macroscopic: 0 absence, 1 presence.

°° Parasites microscopic count: number in the ear washing product.

**Table 3.** Status of the control and treated cats at the beginning of the study.

Day −1	Sex*	Right ear			Left ear		
Control cats		Cerumen	Pruritic reflex	Parasites	Cerumen	Pruritic reflex	Parasites
				Macroscopic search			Macroscopic search
4117	F	0	0	0	1	0	0
6504	F	0	0	0	0	0	0
6506	F	1	0	0	0	0	0
1121	F	0	0	0	0	0	0
9165	M	1	0	0	0	0	0
7448	F	0	0	0	0	0	0
Treated cats		0.33	0/6	0 cat	0.17	0 cat	0
8261	F	0	0	0	1	0	0
7138	F	1	0	0	1	0	0
2823	F	0	0	0	1	0	0
2787	M	0	0	0	0	0	0
6533	M	0	0	0	0	0	0
6532	M	0	0	0	0	0	0
		0.17	0/6	0 cat	0.5	0 cat	0

* Male cats are neutered.

Group 1 cats were the six negative untreated controls mixed with three infested “donor” cats. Group 2 cats were treated topically with Broadline^®^ and mixed with three infested “donor” cats using the commercial applicators and volumes (0.3 mL for cats < 2.5 kg and 0.9 mL for cats > 2.5–7.5 kg). The product was applied in one spot, directly onto the skin, on the neck between the shoulders. Broadline^®^ (fipronil 8.3% w/v, (S)-methoprene 10% w/v, eprinomectin 0.4% w/v and praziquantel 8.3% w/v) is a topical combination developed for cats with the aim of offering a wide spectrum of antiparasitic activity [12]. Its efficacy has been demonstrated against fleas, ticks, roundworms, hookworms and cestodes, but has not yet been evaluated against ear mites.

The cats from each group were kept all together in a 20-m^2^ room for one month (photo 1). The study monitor and investigator were blindfolded, but not the technicians who handled the cats. The technicians changed their clothes between the handling of group 1 and group 2 so as to avoid any transfer of antiparasitic drugs from the treated cats to the non-treated cats. Moreover, to avoid any cross-contamination, group 1 cats were always handled the day before group 2 cats. Cats were followed up clinically as described in Table 1 for 28 days, and both clinical examination and ear mite counts were conducted on Day 28. To check the infestation status at Day 28, a visual otoscopic examination was performed and followed by the cleaning of each ear canal by rinsing it with an ear hygiene liquid. The flushing was repeated three times per ear and the liquid collected and put in a Petri dish in order to be observed microscopically.

All cats were aged more than 6 months and weighed more than 2 kg. They were healthy at the start of the study. Any cat showing an extreme form of adverse reaction or suffering from a life-threatening disease, or extreme discomfort would have been removed from the study.

Food and water were provided in stainless steel bowls, food was given once a day and the water was replenished at least twice daily. The cats were observed hourly for four hours post-treatment for possible adverse events.

The primary assessment criterion was the number of mites recovered from the cats in the control versus the treated group on Day 28. The second criterion was the clinical scoring system based on cerumen abundance and ear pruritus. The cerumen was scored from 0 (absent) to 3 (very abundant) and the ear pruritic reflex from 0 (absent) to 3 (pronounced).

The percentage efficacy for the treatment group for the prevention of *O*. *cynotis* infestation was calculated as follows:

$$\mathrm{Efficacy}\;(\%)=100\times (T_{c}-\;T_{t})\;/T_{c},$$

where:

*T*_c_ = Total number of mites recovered from cats in the negative control Group 1.
*T*_t_ = Total number of mites recovered from cats in the treated Group 2.

The groups were compared using SAS^®^ version 8. The level of significance of the formal tests were set at 5%; all tests were two-sided.

## Results

All donor cats were confirmed to be chronically infested with *Otodectes cynotis* on Day −1 and still on Day 28 (Table 2). A total of 1024 live ear mites were recovered from the donor cats included in the control group 1 (untreated) and 1415 from the donor cats included in the treated group 2; the difference was not significant. For an unknown reason, left ears seemed to be more infested than right ears, although it was not significant (*t*-test > 0.05).

From untreated cats, 129 live mites were recovered on Day 28 and all cats were found to be infested (Tables 3 and 4). In the treated group, three cats were found to be infested, with a total of five mites recovered, the difference between the two groups being significant (*p* = 0.003). One treatment corresponded to 96.12% preventive efficacy at Day 28 based on the primary criterion of ear mite counts.

**Table 4.** Comparison of control and treated cats at Day 28.

Day 28	Sex	Right ear			Left ear		
Control cats		Cerumen	Pruritic reflex	Number of parasites	Cerumen	Pruritic reflex	Number of parasites
				Microscopic°°			Microscopic°°
4117	F	3	2	8	2	1	9
6504	F	2	0	21	1	1	19
6506	F	2	0	12	2	2	6
1121	F	2	1	11	2	1	1
9165	MC	2	1	13	2	2	19
7448	F	2	2	5	2	2	5
	Average	2.17		70	1.83	6/6 cats	59
		6 cats	4 cats	6 cats	6 cats		6 cats
Treated cats	Sex	Cerumen	Pruritic reflex	Microscopic°°	Cerumen	Pruritic reflex	Microscopic°°
8261	F	0	0	0	0	0	0
7138	F	1	0	0	1	0	2
2823	F	1	0	0	0	0	0
2787	MC	0	0	0	0	0	0
6533	MC	1	0	0	0	0	2
6532	MC	1	0	0	1	0	1
	Average	0.67	0	0	0.33	0	5
		4 cats	0 cat	0 cat	2 cats	0 cat	3 cats

Cerumen: 0 = absent; 1 = present but clear and low volume; 2 = moderate volume and brown; 3 = abundant dark.

Ear reflex: 0 = absent; 1 = present and very low; 2 = mild; 3 = pronounced.

°° Parasites microscopic count: number in the ear washing product.

The clinical scoring of the cats in groups 1 and 2 did not show any differences at Day −1. With regard to the criterion of cerumen abundance, the clinical score increased significantly for group 1 cats between Day −1 and Day 28 (*p* = 0.00026) and not for group 2 cats (*p* = 0.30). The difference in cerumen abundance was significant between group 1 and group 2 cats on Day 28 (*p* = 0.0035).

Concerning the clinical criterion of pruritic reflex in at least one ear, all cats were negative at inclusion (no ear pruritus). All six control cats showed a pruritic reflex on Day 28, whereas no treated cat showed ear pruritus (*p* = 0.00026).

## Discussion

The infestation model was successful as all control cats were infested with *Otodectes cynotis* after 28 days of direct contact with donor cats. Only three infested cats enabled the infestation of six receiver cats. This is a demonstration of the direct transmission of *Otodectes cynotis* during social activity of cats, which certainly happens in real life. All cats were older than 6 months, meaning that ear mange is not only a kitten dermatitis and that adult cats may transmit it and express clinical symptoms [9].

One administration of Broadline^®^ controlled the development of clinical signs of ear mange and reduced the number of mites passing from donor cats to receiver cats by 96.12%. The respective effectiveness of eprinomectin or fipronil is unknown, both having acaricidal activity. Fipronil has been shown to be active against ear mites when administered inside the ear canal [4, 7]. No data are available for eprinomectin. Nevertheless, other avermectins/milbemycins, i.e. moxidectin and selamectin, have demonstrated their efficacy against *Otodectes* mites [2, 5, 11]. It can be hypothesized that both fipronil and eprinomectin may act in this topical formulation. Fipronil, which acts topically by direct contact, probably acts during the migration of the mites onto the skin to reach the ear canals. Eprinomectin, which is active systemically, probably acts on *Otodectes* when they are inside the ears and bite to feed [8].

The experimental model requires the flushing of the ears to count the mites as the otoscopic evaluation is not reliable. It means that the study could not be continued after Day 28 as all mites were removed. This model should probably be used for longer periods, to study prevention against regular risk. In such a case, it is probable that regular monthly administrations would provide complete control of the risk.

## Conflict of interest

The work reported herein was funded by Merial S.A.S., France. The authors are current employees or contractors of Merial.

^®^Broadline is a registered trademark of Merial. All other brands are the property of their respective owners.

This document is provided for scientific purposes only. Any reference to a brand or trademark herein is for informational purposes only and is not intended for a commercial purpose or to dilute the rights of the respective owner(s) of the brand(s) and trademark(s).
